# 3D Printing of Molecular Models

**DOI:** 10.5210/jbc.v40i1.6626

**Published:** 2016-02-26

**Authors:** Adam Gardner, Arthur Olson

**Affiliations:** 1 The Scripps Research Institute

## Abstract

Physical molecular models have played a valuable role in our understanding of the invisible nano-scale world. We discuss 3D printing and its use in producing models of the molecules of life. Complex biomolecular models, produced from 3D printed parts, can demonstrate characteristics of molecular structure and function, such as viral self-assembly, protein folding, and DNA structure. Advances in computer and user interface technology have enabled physical molecular models to combine with augmented reality to bridge the physical with the computational world.

## Introduction

Physical models have had a long and important role in exploring and communicating scientific and medical concepts and structures. One of the earliest physical scientific visualizations was the orrery, a mechanical model of the solar system, which first appeared in Greece around 150 BC. Early anatomical models, such as those made from papier-mâché were developed in the early-19th century. And, the earliest molecular models were those produced in the mid-19th century by chemists such as Hoffman and Kekule to visualize the nature of chemical composition. These models brought abstract or unseen structures into the context of human experience.

Physical models convey spatial relationships and mechanisms in ways that images alone cannot. They engage perceptual and cognitive processes that go beyond the visual, and bring a sense of reality and natural interaction into the process of exploration and understanding. In the context of molecular science, they create a human-scale, tangible representation to objects that are too small to be directly perceived. Physical molecular models can also serve as “analogue computers,” where the spatial relationships between the components involved in complex molecular interactions can be explored and manipulated. The prime example of this utility was in the discovery of the structure of DNA. Watson and Crick manipulated models of the nucleotide bases, whose structures and dimensions were already known from chemistry, to develop the double helical model of base-pair complementarity, that explained the molecular mechanism of genetic inheritance, underlying all of biology.

Throughout the first half of the 20th century, physical molecular models were ubiquitous in chemical laboratories and educational institutions. The advent of interactive computer graphics in the 1960’s and 70’s eventually replaced such models with images on a computer screen. The power and unlimited variability of a computer model had significant advantages over the fixed nature of a physical model. This was especially true for complex biological molecules, which were very time consuming and difficult to accurately build as physical models. On the other hand, the real-world physical models had characteristics, as mentioned above, that provided advantages that the computer images lacked. The advent of computer auto-fabrication, now referred to as solid or 3D printing, provided an automated way to produce physical objects from computer data, and opened up the possibility of having highly accurate, material visualizations of complex molecules and their assemblies. Here we discuss the nature of 3D printed molecular models, how they are produced, and what they can provide for visualization and communication.

## Methods

### 3D Printer Basics

3D printing is an additive manufacturing process in which an object is produced layer by layer from a digital geometric description of a model. The computer model must describe the boundaries of the object in such a way that there is an unambiguous distinction between what is inside and what is outside of the object. This computer model is sliced into planar sections, so that a 3D printer can reconstruct the model by depositing or fusing material for each successive section. While there are a growing number of different types of 3D printers, with a wide variety of mechanisms and materials, currently all 3D printers operate in this way. The fact that the output from a 3D printer is a physical object, dictates that there are both material and physical constraints inherent in the production and subsequent use of the printed object. These constraints depend upon the nature of the 3D printer and the material used. The characteristics of the printing process and output model, such as speed of production, strength, precision, durability, and color are important to take into consideration in the conception, design, and production of any 3D printed model.

The most common 3D printers on the market today can be classified by the kind of material feedstock that they require. The first 3D printers to come on the market used photo activated liquid resins. These resins solidify when exposed to light of a specific wavelength. An object can be “printed” by a laser, scanning successive slices of the object on a platform that descends, step-by-step in the liquid resin. After the final “top” slice is scanned, the platform rises out of the surrounding liquid resin, exposing the solidified object. Two issues that are common to many 3D printing techniques arise in this process. First, as an arbitrarily shaped object is being printed layer-by-layer, there may be parts of the object that have nothing below it in the previously printed layer. Thus, there must be some support structure designed into the process that is built along with the object being printed. In these resin-based printers, the support structure is printed from the same resin, but designed to be broken away after the printing is finished. Secondly, many of the 3D printing methods require some “finishing” or post-processing. In the case of the early resin printers, ultraviolet light hardening or curing was required prior to the manual removal of the supporting structure. The finished product from the resin-based printer has the appearance of a piece of transparent amber. Little or no color variation could be attained in these early resin-based printers. Today there are more advanced resin-based printers that can deposit multiple, different, photosensitive materials onto each section, enabling a mixture of material characteristics and expanded color variation.

**Figure:**
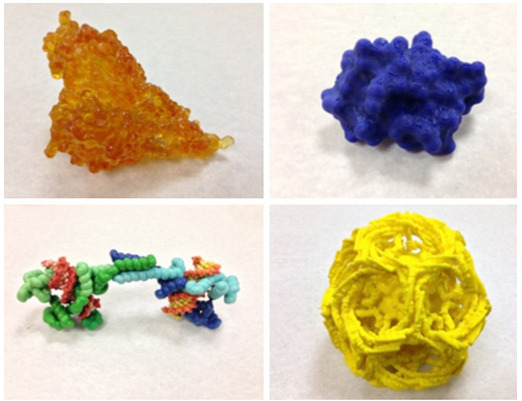
*** Figure 1*** . ***3D printed molecular models from different printers:*** *A. Bacterial pilin assembly printed by StereoLithography (light activated resin); B. DNA condensing protein printed by Z-corp powder-based full color printer; C. Crambin protein printed on hobbyist FDM printer (ABS plastic); D. Clathrin-coated vesicle printed on professional FDM printer (ABS plastic).*

Another type of 3D printer uses powder or fine granules of material. The first commercially available, full color, 3D printers use gypsum (plaster of Paris) powder. Color binder “inks” are applied with inkjet printer heads onto each successive layer of the powder after it is spread on a descending platform. For full color, four print heads with cyan, yellow, magenta and clear binder are used. The white unbound powder remains loose and serves as a support for subsequently built layers of the object. Thus, when the printing is finished, the printed object lays buried in the loose powder and it must be carefully excavated and any remaining loose powder must be removed. Since the inkjet print heads cannot utilize strong glues, the object is porous and fragile. It must be carefully removed from the printer to be post-processed in order to strengthen it by infusing a stronger binder, such as wax or superglue. Other granule-based printers use laser sintering to heat fuse the object layer by layer. The sintering granules can range from nylon to metal. These objects are quite strong when they are removed from the loose powder, but they are monochrome, and if color is desired, it must be applied afterward – typically by dyeing or hand painting.

Another popular class of commercially available 3D printers uses a process known as fused deposition modeling (FDM). These printers utilize plastic filament feedstock which is fed from a reel through a computer controlled XY linear axes moving heated nozzle and extruded as a fine thread scanning sections of the model, layer by layer onto a platform that moves in Z axis. Like the resin printers, support material must also be deposited in each layer to underpin any model overhangs in the layers that follow. The feedstock is typically a single color, but some printers come with multiple heads for multiple materials, including different colors or soluble support material.

The cost of 3D printers varies widely, depending on the nature of the printing mechanism, its reliability and precision, as well as the size of the printer’s build volume and material characteristics of the feedstock. Multi-material photo activated resin printers and laser sintering printers can cost upward of hundreds of thousands of dollars, while the extruded plastic printers can be had for as little as $1000, and are trending toward becoming consumer “appliances.” While owning a 3D printer may now be widely achievable, there also exists an increasing number of 3D printing services, to provide greater capabilities and wider material choices that are not otherwise available to the consumer. The cost of a 3D printed object is typically a function of the time on the printer, the cost of the feedstock materials, as determined by the size of the printed object, and by the human time that is required in the setup and post processing of the piece

**Table 1:** Some commercially available 3D printers (Consumer and Professional)

**Consumer (Hobbyist)**							
**Printer**	**Manufacturer**	**Class**	**Materials**	**Support**	**Material Color**	**Material Cost**	**Machine Cost**
RepRap	Open Source	FDM	Multiple	Plastic Rafting	1 - 3+	Variable	Variable
Makerbot	Makerbot Industries	FDM	PLA, ABS, Flexible	Plastic Rafting Dissolvable Support	1 - 2	Low	$1,375 - 6,499
Form1	FormLabs	Stereo- lithography	Photo Activated Resins	Hanging Support	Clear, White, Grey, Black	Medium	$3,200
CubeX	Stratasys	FDM	ABS	Plastic Rafting	1 - 3	Medium	$2,499 - 3,999
**Professional**							
Dimension	Stratasys	FDM	ABS	Dissolvable Support	1	High	$35,000- 70,000
ProJet (Z Corp)	3D Systems		Plaster	Self-Supporting	Full Color	High	$25,000- 80,000
Objet	Stratasys	Sprayed Photopolymer	Photo Activated Resins	Wax Support	Selected Color	High	$30,000- 150,000
Mcor	Mcor Technologies	Fused Paper	Paper	Self-Supporting	Full Color	Low	$35,000 - 50,000

### 3D Printing of Molecular Models

Molecules are composed of atoms, bonded together into a specific connectivity and 3D geometry. Biological molecules, such as proteins, carbohydrates, and nucleic acids can be composed of hundreds to thousands of atoms. The principal source for atomic coordinates of biomolecules is the Protein Data Bank, which currently holds over 100,000 entries. These entries are in the form of the atomic coordinates of the molecular structures. As such, they contain only the three dimensional points in space (the X, Y, Z axes positions) of the atoms and their chemical types. To turn that information into a buildable 3D object, the first task is to decide on a representation in which to render the molecular structure. It should be noted that the molecular images that are rendered on a computer screen do not necessarily translate well into a solid physical model, since both the nature of the 3D printer and the geometry and mechanical characteristics of the real-world model can defeat the change in medium.

Specifying all of the atoms in the molecule as spheres of characteristic atomic radii – the so-called CPK (Corey-Pauling-Kultun) model -- is the simplest representation, but has some drawbacks, from the 3D printing perspective. Each sphere is typically represented by a triangular polygonal mesh. Thus, CPK models of protein structure are composed of a very large number of polygons, many of which intersect with each other. This representation can create very large print files. Additionally, most of these atomic spheres are internal to the protein and are not visible in the physical model, and the packing of these spheres leaves numerous small vacant spaces, from which it may be difficult to remove powder or other support material.

Another popular computer graphic molecular representation is the protein backbone ribbon model, which shows the molecular “skeleton” or 3D fold. Since this representation is essentially a meandering curvilinear structure it may need extra bolstering (e.g. struts). This depends upon the strength and characteristics of the printing material used, so that the model is robust enough to build and handle without breakage. Plastic and resin models are more robust than the full color gypsum-based models for this type of representation. However, even in plastic, FDM models with long, thin rod-like sections may be quite weak. Moreover, the orientation of the model in the build can determine the strength of the finished part – rods printed parallel to the section direction are much stronger than those aligned perpendicular to it.

The third class of molecular representations depict the molecular surface, or skin, -- that part of the molecule that can come in contact with the solvent environment (usually water). Several molecular modeling codes provide for the calculation of the solvent accessible (or solvent excluded) molecular surface, which provides an atomic resolution description of such a surface. In addition, lower resolution molecular surfaces can be used to represent the shape of the molecule at a user-defined level of detail. They are typically isocontour surfaces of a volume, derived from Gaussian functions placed at atomic centers. Molecular surfaces are ideal for representing the shape of large biological molecules for 3D printing, since they have a lower polygon count and are typically compact globular structures, needing no additional bolstering for mechanical strength. Additionally, surface models can be built with hollow or sparsely filled interiors, using less material and being lighter to handle.

**Figure 2:**
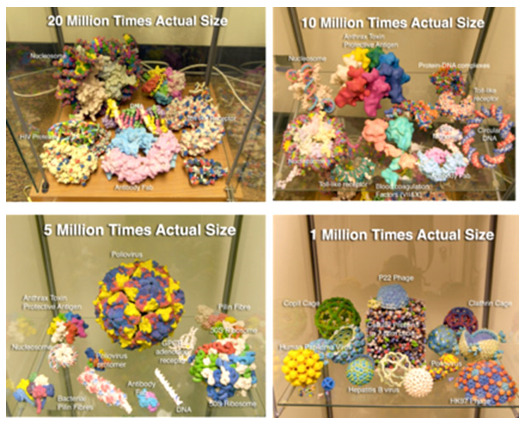
** 3D printed molecular models at various scales.** Full color models are made with the powder-based Z Corp printer. Monochrome models are made with the FDM Stratasys printer

Scale is an important consideration when printing molecular models. The size of a tangible molecular model is millions of times the size of the actual molecular structure that it represents, however it is limited by its convenience to the human user and by the physical constraints of the 3D printer. The size of actual molecular structures can range from a small number of atoms in simple chemical compounds (about 1 nanometer across) to many thousands or millions of atoms in large molecular assemblies, such as ribosomes and viruses 10s to 100s of nanometers in diameter. Thus, a single scale for all tangible molecular models is impractical. Yet, the use of a set of specific scales for molecular models proves to be beneficial to enable model comparisons and to explore interactions between them. Using an arbitrary scale for a molecular model may be suitable for isolated, large sculptural objects, but it limits the utility of hand held models. We tend to use 40 million times actual size for models made up of atomically-manipulable components, 20 million times actual size for individual proteins; 10 million times for protein assemblies with several interacting protein chains; 5 million times for very large molecular assemblies and viruses; and 1 million times for larger cellular components and structures. These scales produce tangible models that can be easily held and manipulated in the hand (Figure 2 ).

### Software for 3D Printed Molecular Models

The most common computer formats accepted by 3D printers are: STL, OBJ, X3D, Collada or VRML. Many molecular modeling packages now provide output formats (usually STL or VRML) that are compatible with 3D printers (Table 1). Sending a file in one of these formats to a 3D printer, however, does not guarantee that it is physically buildable. As stated above, the file must represent an enclosed volume, termed a “manifold geometry”. A file with a list of polygons may have open edges that do not enclose a volume, or polygons may have surface normals, that are pointing inside rather than outside the object. Molecular modeling programs do not typically analyze for these, but there are several programs that can repair such non-manifold geometries, e.g. MeshLab (http://meshlab.sourceforge.net ). While the geometries generated from these molecular modeling programs may be directly 3D printable, it does not guarantee that the model is mechanically strong enough to hold together or be handled. Additionally, one might desire mechanistic features that go beyond the static shape of the model. For example, one might want to add extra features such as bolstering or other types of affordances that go beyond the functionality of the molecular modeling software. For these types of features, one must use more generic computer-aided design software. Most of the high-end 3D modeling and animation packages now used by medical and scientific illustrators, such as Maya, 3D Studio Max, Cinema4D, and Blender have sufficient modeling capabilities to modify input molecular geometries with other constructed geometries and support operations such as Boolean addition and subtraction of volumes for the desired finished model geometry. We have produced a molecular modeling plugin for these 3D modeling and animation packages, called ePMV (embedded Python Molecular Viewer), that enables the construction of molecular representations inside these high end codes, eliminating the need to transfer information between the molecular modeling environment and the more generic modeling environment.

### Examples of 3D Printed Biomolecular Models

The shapes and chemistry of biomolecules dictate their function. 3D printing enables the representation of these characteristics in a tangible and easily grasped form. While complex shapes can be produced by most of the current 3D printers, representing the chemical nature of the molecules can be more problematic. Color has long been used by chemists to indicate different atom types (e.g. red oxygen, blue nitrogen, black carbon, etc.). This type of color-coding has carried through to distinguish other aspects of complex molecules, such as amino acid type, protein chain identity and electrostatic potential. Of the 3D printers currently on the market, there are only a few that can create full color models. These include the Z Corp powder-based printer, and the Mcor laminated paper printer. While plastic extrusion printers can use different color filament, and some with multiple heads can incorporate more than one color into a single object, their ability to mix colors within a layer are very limited, at best. If the molecular model is composed and built from multiple separate parts, then different color plastic can be used to identify these different parts. Examples of full color molecular models printed on the Z Corp printer and multiple color assembled plastic models are shown in Figure 1.

**Figure 3:**
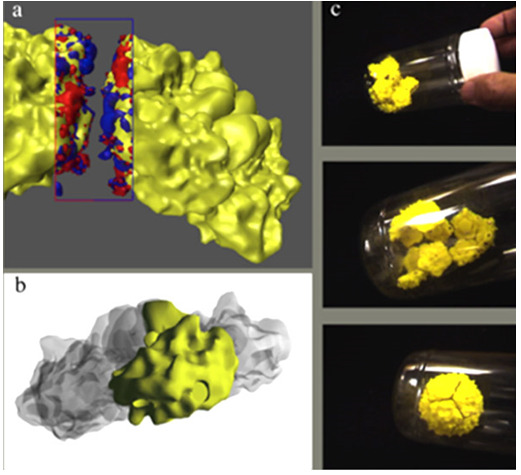
(a) Two 5-sided poliovirus subunits showing complementary electrostatic forces between them. (b) Geometry used to make the subunit. (c) Sequence showing self-assembly of 12 subunits into a complete virus capsid.

3D printing can be used to produce molecular models that demonstrate function, as well as shape. An example of such a model that we have developed demonstrates the process of self-assembly of virus capsids. This model is based upon the atomic resolution structure of the poliovirus (Figure 3 ).

The capsid assembles in a hierarchical fashion; wherein five fundamental building blocks, composed of four different proteins, first form into a pentagonal structure, 12 of which then assemble into the intact spherical capsid. We modeled the last step of this assembly process by printing surface models of the 12 identical pentamers using an FDM plastic printer. In the virus, the edges of these pentameric subassemblies have complementary electrostatic charges and shapes that facilitate their assembly. We used magnets, imbedded into the edges of the model, to mimic this complementarity. In order to place cylindrical magnets in the proper locations along each edge, we used a generic computer aided design (CAD) package to Boolean subtract cylindrical holes from our molecular surface model. Subsequent to printing these models, we placed and glued the cylindrical magnets, with the proper orientation, into the pre-made holes, such that the pentamers could attract one another. Placing 12 of these pentamers into an enclosed container and shaking with the proper energy demonstrates the process of self-assembly, as the individual pentamers ultimately find and bind to each other to form a complete spherical capsid (Figure 3 ). This model has been used and tested in a number of educational settings to teach the concept of self-assembly from random motion.

Many other aspects of biological function can be demonstrated using models composed of 3D printed parts. The folding of a protein from a linear polypeptide chain of amino acids is fundamental to attaining the shape of the functional molecule. Using the known geometry of the peptide backbone we printed a Lego-like set of parts, designed to assemble into a chain which can be folded into a given protein shape. In order to do this, we imbedded magnets into the peptide unit to mimic the hydrogen bonding that forms the structural backbone (secondary structure) interactions. We imparted geometric flexibility to the hydrogen bonding by in situ printing of a ball and socket joint for the hydrogen bond acceptor to represent its known angular interaction range. Since the orientation of each amino acid in the chain is related by two rotational angles (phi and psi) to its two adjacent amino acids, we encoded these angles into the base (alpha carbon) of each by specifying a complementary fit to each flanking peptide unit.

**Figure 4:**
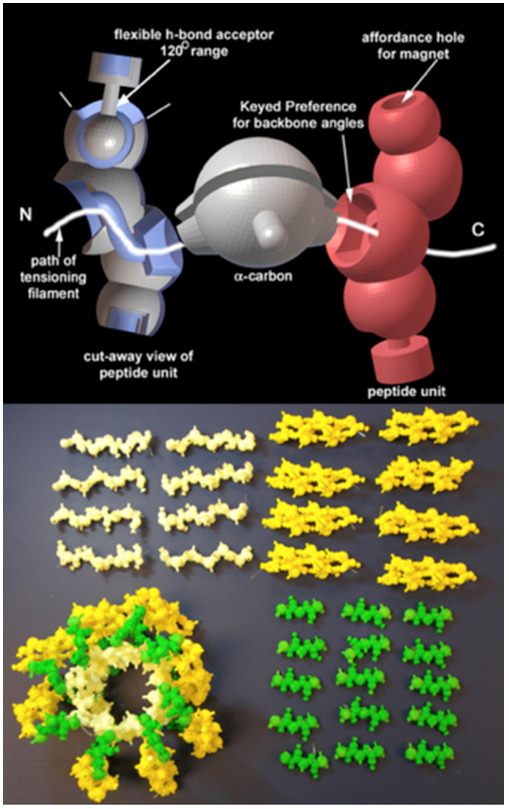
Protein assembly kit. Above, computer model showing flexible ball-socket and preference keyed phi/psi sockets. Below, protein folding components and assembled protein structure.

The peptide units and the base alpha carbons are strung successively on an elastic monofilament, such that the tension on the filament drives the chain into the preferred specific folding configuration. Thus, while the peptide chain is free to rotate at each amino acid, there is a tangible fit when the angle of rotation is at the preferred value. Since the major structural motifs of proteins consist of alpha helices and beta sheets, with canonical phi-psi angles, we have made units of such motifs along with loop structures with no preferred phi-psi angles, that can be snapped together to make a generic protein folding kit. Because of the accurate protein geometries built into these models, they demonstrate some of the emergent properties inherent in the building up of protein structures. We have used these kits in educational settings that range from high school to graduate school (Figure 4 ).

**Figure 5:**
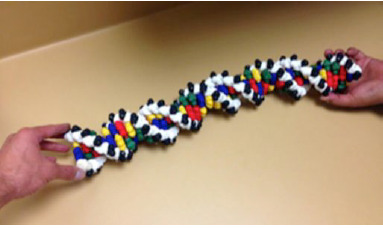
Manipulable Flexible DNA Model. 3D printed components. The reconfigurable model has sugar, phosphate and nucleotide components that can be assembled into arbitrary sequences and lengths of the individual strands.

**Figure 6:**
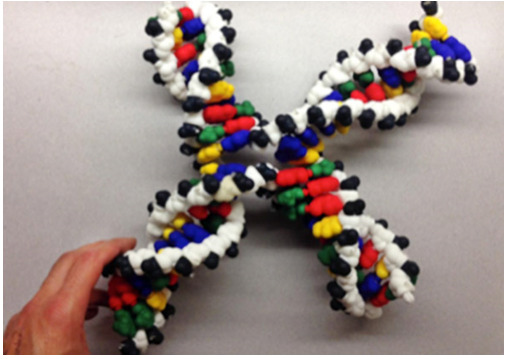
DNA Model configured as a Holliday junction   demonstrating strand exchange between 2 double helices.

We have extended this component-based approach to creating a flexible DNA model to demonstrate the nature of double helix, base-pair complementarity, templated replication and other DNA configurations. In this model the four nucleotide bases are printed in a different color on an FDM plastic printer. Similar to the polypeptide backbone folding model, the Watson-Crick hydrogen bonds are mimicked by imbedded magnets. The DNA backbone is created with two kinds of FDM printed plastic parts, the sugar and the phosphate (white and black, respectively). The sugar has a snap-bead fitting, designed to mate with any of the nucleotide bases. The sugar and phosphate units are strung, using elastic monofilament to create the DNA backbone. Similar to the polypeptide backbone the phosphate has angles encoded into it that guide preference for the B-form DNA helical structure. The DNA model components are either strung as five base units to form a helical half-turn, or as individual nucleoside units, to enable demonstration reaction of nucleotide triphosphate for base addition via DNA polymerase. The units have “pop-bead” snap fits at the 5’ and 3’ end to enable extension of the DNA strands and other DNA structures, such as those involving strand exchange between two double helices (Figures 5 & 6). This model is currently being tested in high school biology classes.

## Conclusion

As medical science becomes more molecular, the ability to perceive, explore and understand molecular structures and their interactions become more important for students, medical practitioners, scientists, and the lay public. Physical models have a power and presence that is lacking from pictures, animations, and even interactive computer graphics. The advent of solid 3D printers has democratized the production of custom physical models and extended their production and application to models of the unseeable molecular world. The field of 3D printing is still in its infancy, with new types of printers, materials, and capabilities changing rapidly. The field of bioprinting biocompatible scaffolds, and even cellular structures, is one area of great current interest. 3D printing of complete devices that include power supplies, circuits and actuators is in the near, foreseeable future.

**Figure 7:**
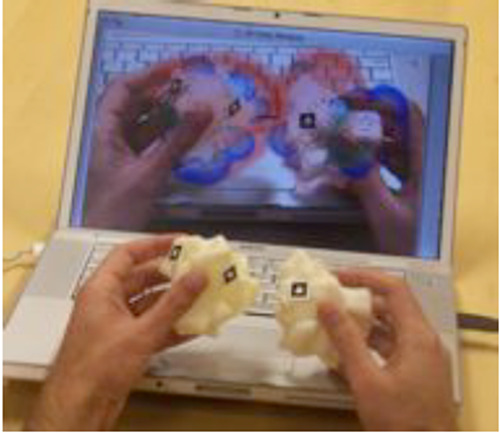
Augmented reality tangible molecular interface showing manipulation of two 3D printed physical models of subunits of the enzyme Cu-Zn superoxide dismutase, augmented on the computer screen with the electrostatic potential fields that are computed for the two subunits.

**Figure 8:**
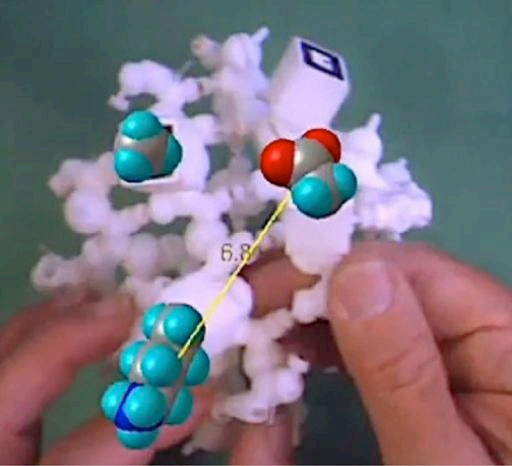
Tangible molecular interface comprised of 3D printed polypeptide backbone model of a beta barrel, augmented with virtual amino acid side chains and interactive distance computation between two amino acids.

For the purposes of visualization, 3D models can be integrated with the computer models and molecular information from which they were modeled using the technology of augmented reality. We have been developing what we term “tangible molecular interfaces” which combine the manipulation of physical molecular models with computer visualization and calculation (Figures 7 & 8 ). As tracking and computer display technology progresses, the ability to use physical manipulation of real object will couple seamlessly with the computational environment. We see a future where of biomedical visualization brings us closer to the molecular world in which we live. 3D printing will be part of that future.
